# Endoplasmic reticulum stress-related neuroinflammation and neural stem cells decrease in mice exposure to paraquat

**DOI:** 10.1038/s41598-020-74916-x

**Published:** 2020-10-20

**Authors:** Zhengli Yang, Yiming Shao, Yifan Zhao, Qian Li, Rui Li, Hongxi Xiao, Fen Zhang, Yilan Zhang, Xiuli Chang, Yubin Zhang, Zhijun Zhou

**Affiliations:** 1 School of Public Health/MOE Key Laboratory of Public Health Safety/NHC Key Lab of Health Technology Assessment, Fudan University, 130 Dong’an Road, Shanghai, 200032 China; 2 Pharmacology and Toxicology Department, Shanghai Institute for Food and Drug Control, Shanghai, 201203 China; 3 Institutes of Brain Science, State Key Laboratory of Medical Neurobiology, Fudan University, Shanghai, 200032 China

**Keywords:** Public health, Neuroscience, Risk factors

## Abstract

Paraquat (PQ), a widely used herbicide, could cause neurodegenerative diseases, yet the mechanism remains incompletely understood. This study aimed to investigate the direct effect of PQ on NSC in vivo and its possible mechanism. Adult C57BL/6 mice were subcutaneously injected with 2 mg/kg PQ, 20 mg/kg PQ or vehicle control once a week for 2 weeks, and sacrificed 1 week after the last PQ injection. Furthermore, extra experiments with Tauroursodeoxycholic Acid (TUDCA) intervention were performed to observe the relationship between ER stress, neuroinflammation and the neural stem cell (NSC) impairment. The results showed that 20 mg/kg PQ caused the NSC number decrease in both subgranular zones (SGZ) and subventricular zone (SVZ). Further analysis indicated that the 20 mg/kg PQ suppressed the proliferation of NSC, without affecting the apoptosis. Moreover, 20 mg/kg PQ also induced ER stress in microglia and caused neuroinflammation in SGZ and SVZ. Interestingly, the ER stress inhibitor could simultaneously ameliorate the neuroinflammation and NSC reduction. These data suggested that increased ER stress in microglia might be a possible pathway for PQ-induced neuroinflammation and NSC impairment. That is a previously unknown mechanism for PQ neurotoxicity.

## Introduction

Paraquat (PQ), a widely used non-selective herbicides in the world, is known for its potent neurotoxicant properties^1^. Indeed, the PQ toxicity has been widely discussed but is not yet fully clarified. The underlying mechanism may be associated with mitochondrial dysfunction, lipid peroxidation, and oxidative stress etc.^2–5^.

In line with these data, epidemiological studies reported that PQ could result in cognitive and behavioral impairments and increase the risk of neurodegenerative diseases, such as autism spectrum disorders and Parkinson’s disease^6–8^. In this context, experimental evidences showed that PQ exposure could cause permanent nerve damage in mice^9,10^. NSCs in the adult mammalian brain are regarded as a useful tool to regenerate neurons in pathological conditions such as neurodegenerative diseases or acute brain injury, indicating that the NSC impairments may be closely correlated with the nervous system diseases^11^. It was reported that exogenous hNSCs could improve the behavioral function in the model of Parkinson’s disease. Actually, as most primordial cell in the central nervous system, the NSC appeared to promote function preservation and/or restoration in neurodegenerative disease^12^. Besides, PQ-induced functional impairments in progenitor cell in vitro were also reported^13–15^. However, to date, there is no study evaluating the direct toxicity of PQ to NSC in vivo.

In the adult rodent brain, NSCs persist in the SGZ and SVZ, which are specialized niches with a particular architecture, allowing NSCs to survive, proliferate and generate new neurons^16^. NSCs generate the central nervous system (CNS) during development and persist in adult brains at the SGZ and SVZ to make and replenish differentiated cells lost during stress and injury^17–19^. Most CNS injuries elicit proliferation and migration of the NSCs toward the injury site, indicating the activation of a regenerative response. However, regeneration from NSC is incomplete, which could be due to detrimental cues encountered during inflammation^20^. Different CNS diseases can cause activation of the innate and adaptive immune responses that could influence the NSCs. Furthermore, NSCs in the brain react differently to inflammatory cues than their counterparts in the spinal cord^20^. Thus, it is necessary to study the relation between PQ-induced inflammatory and NSC in the brain.

ER stress is a buildup of unfolded or misfolded proteins, which often happened when secreted and membrane-embedded protein translation increased or protein folding decreased in the ER lumen. In response to ER stress, the ER has evolved a capacity termed Unfolded Protein Response (UPR), which could alleviate ER stress by degrading unfolded and misfolded proteins^21^. The canonical UPR signaling is initiated by activation of three ER membrane-bound transducers: Inositol Requiring Enzyme 1 (IRE1), Activating Transcription Factor 6 (ATF6), and Protein Kinase-like ER kinase (PERK)^21^. The relationship between ER stress response and inflammation has gained a massive focus in biomedical research^21^.

Regarding the PQ toxicity to NSC, numerous studies discussed its possible mechanism, but most experiments were based on in vitro designs. Thus, the influences of PQ on NSC in particular regions in vivo remain incompletely defined. To date, the relationships between PQ-induced ER stress and neuroinflammation, NSC impairments are unknown. In this study, we especially focused on SGZ and SVZ where NSCs persist and found that PQ-induced NSC impairment and neuroinflammation, could be restored by an ER stress inhibitor–Tauroursodeoxycholic Acid (TUDCA)^22,23^.

## Results

### PQ reduced the number of NSC in SGZ and SVZ

**Figure 1:**
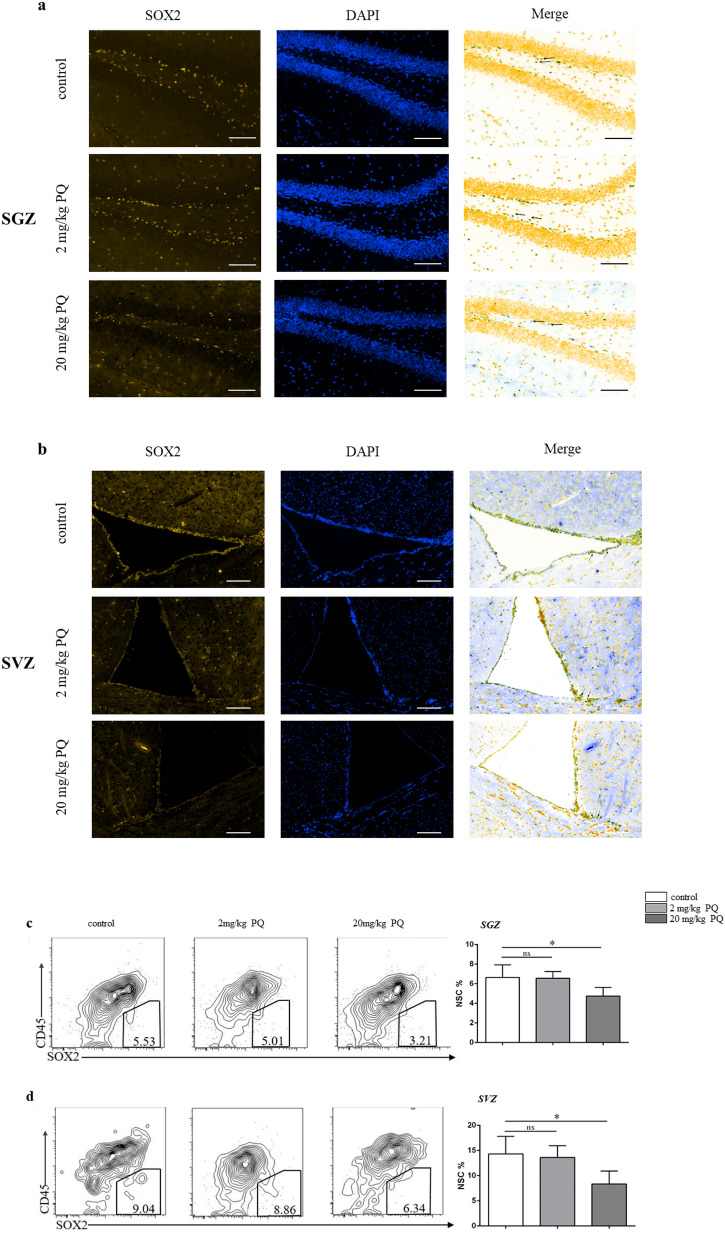
PQ reduced the number of NSC in SGZ and SVZ. B6 mice were treated with PQ twice with 1wk apart and sacrificed 1 week after the last PQ injection. The cell suspensions and slices were prepared from SGZ and SVZ to detect the NSC by flow cytometry or immunohistochemistry staining. **a**, **b** Representative confocal images of NSC (DAPI^+^SOX2^+^) in SGZ (**a**) and SVZ (**b**); DAPI and SOX2 were in blue and yellow, respectively; the representative images of NSC were marked by the black arrows; all the black-green dots represent the NSC; scale bars = 50 μm. **c**, **d** Representative flow plots (left) and quantification (right) of NSC (CD45^−^SOX2^+^) in SGZ (**c**) and SVZ (**d**). Asterisk (*) indicates a significant difference (*p < 0.05, one-way ANOVA) and the n.s. means no difference as indicated. 6 mice were used for each group.

To investigate the effects of PQ on NSC, the immunohistochemical experiments were performed to detect the NSC in SGZ and SVZ. As shown in Fig. 1a, b, 20 mg/kg PQ decreased the number of NSC (marked by the black arrow). Next, using CD45 and SOX2 antibodies^24^ in flow cytometry analysis, we quantified the percentage of NSC. Consistently, the results confirmed the reduction in NSC of SGZ and SVZ (Fig. 1c, d).

### PQ influenced the proliferation but not apoptosis of NSC in SGZ and SVZ

**Figure 2:**
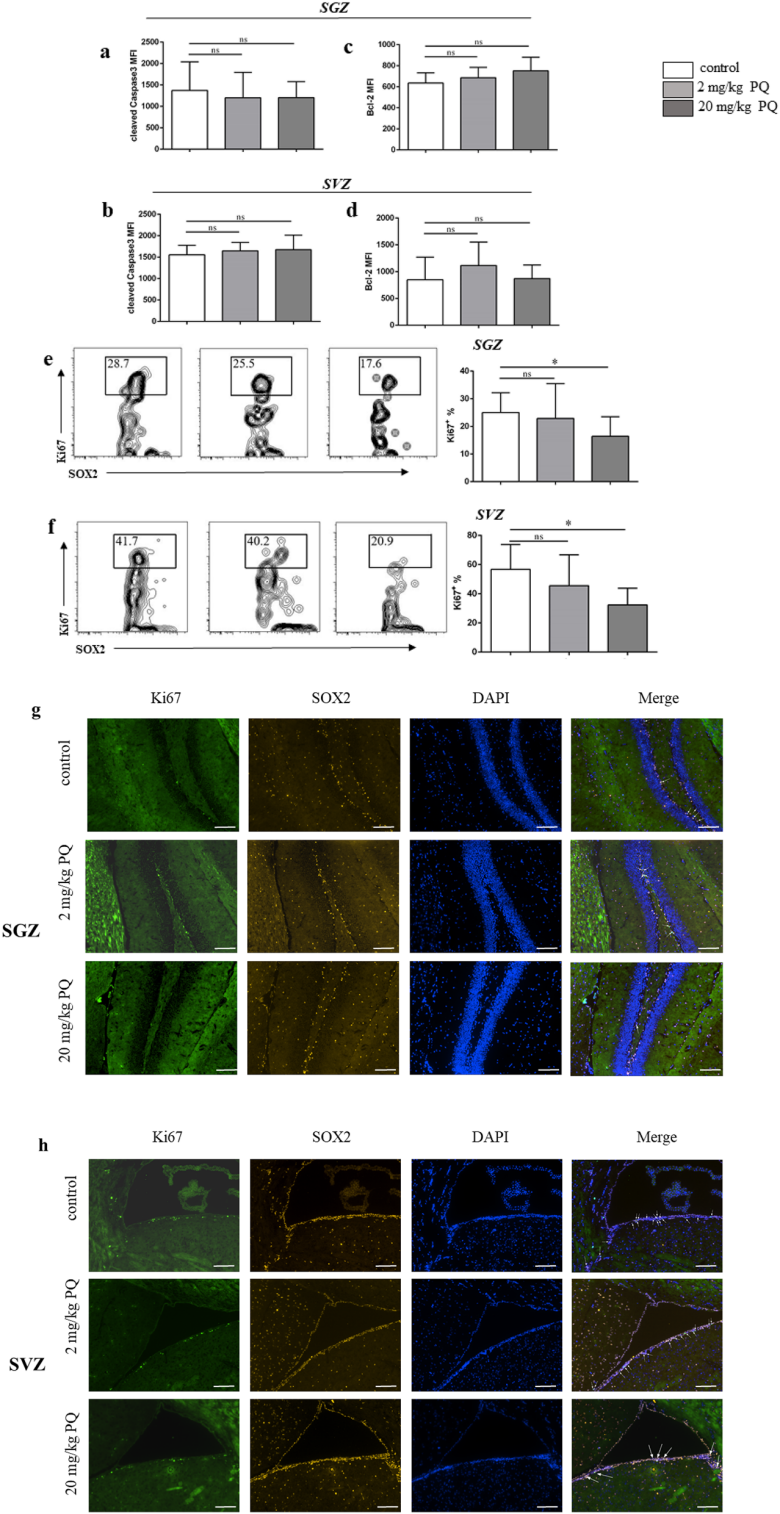
PQ influenced the proliferation, but not apoptosis of NSC in SGZ and SVZ. B6 mice were treated with PQ twice with 1wk apart. Thereafter, the cell suspensions from SGZ and SVZ were prepared to detect the expression of cleaved (active) Caspase-3, Bcl-2, and Ki67 by flow cytometry. The slices were prepared for immunohistochemistry staining. **a**, **b** The mean fluorescence intensity (MFI) of cleaved Caspase-3 of NSC in SGZ (**a**) and SVZ (**b**). **c**, **d** The MFI of Bcl-2 of NSC in SGZ (**c**) and SVZ (**d**). **e**, **f** Representative flow plots for the proliferation (Ki67^+^) of NSC in SGZ (**e**) and SVZ (**f**). **g**, **h** Representative confocal images of proliferation of NSC (SOX2^+^Ki67^+^) in SGZ (**g**) and SVZ (**h**); Ki67, SOX2 and DAPI were in green, yellow and blue respectively; SOX2^+^Ki67^+^cells were marked by white arrows; scale bars = 50 μm. Asterisk (*) indicates a significant difference (*p < 0.05, one-way ANOVA) and the n.s. means no difference as indicated. 6 mice were used for each group.

As the decrease of NSC was observed in SGZ and SVZ from mice treated with 20 mg/kg PQ, we hypothesized that the reduction was due to increased apoptosis or suppressed proliferation. To test this, we firstly measured the apoptosis of NSC in SGZ and SVZ, based on the cleaved (active) Caspase-3, a molecule driving cellular apoptosis^25^. We found that PQ did not influence the apoptosis of NSC (Fig. 2a, b). Further analysis indicated that PQ also did not affect the expression of B-cell lymphoma-2 (Bcl-2) of NSC in the SGZ and SVZ. Bcl-2 is an anti-apoptotic protein that could prevent cellular apoptosis^26^. Next, we further tested the proliferation of NSC in SGZ and SVZ, based on their nuclear expression of Ki67^27^. The results showed that 20 mg/kg PQ inhibited the Ki67 expression of NSC in both zones (Fig. 2e, f). Moreover, immunohistochemistry staining also confirmed the suppressed proliferation of NSC in SGZ and SVZ (marked by white arrow) (Fig. 2g, h).

### PQ activated microglia and induced neuroinflammation in SGZ and SVZ

**Figure 3:**
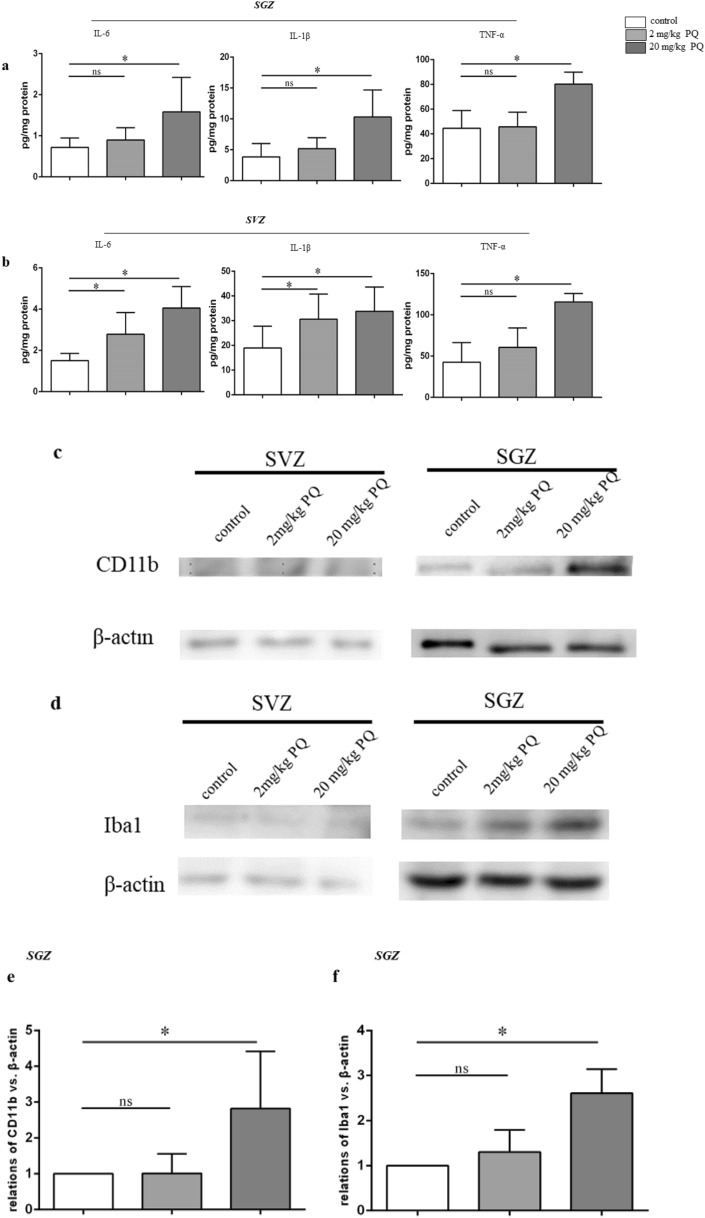

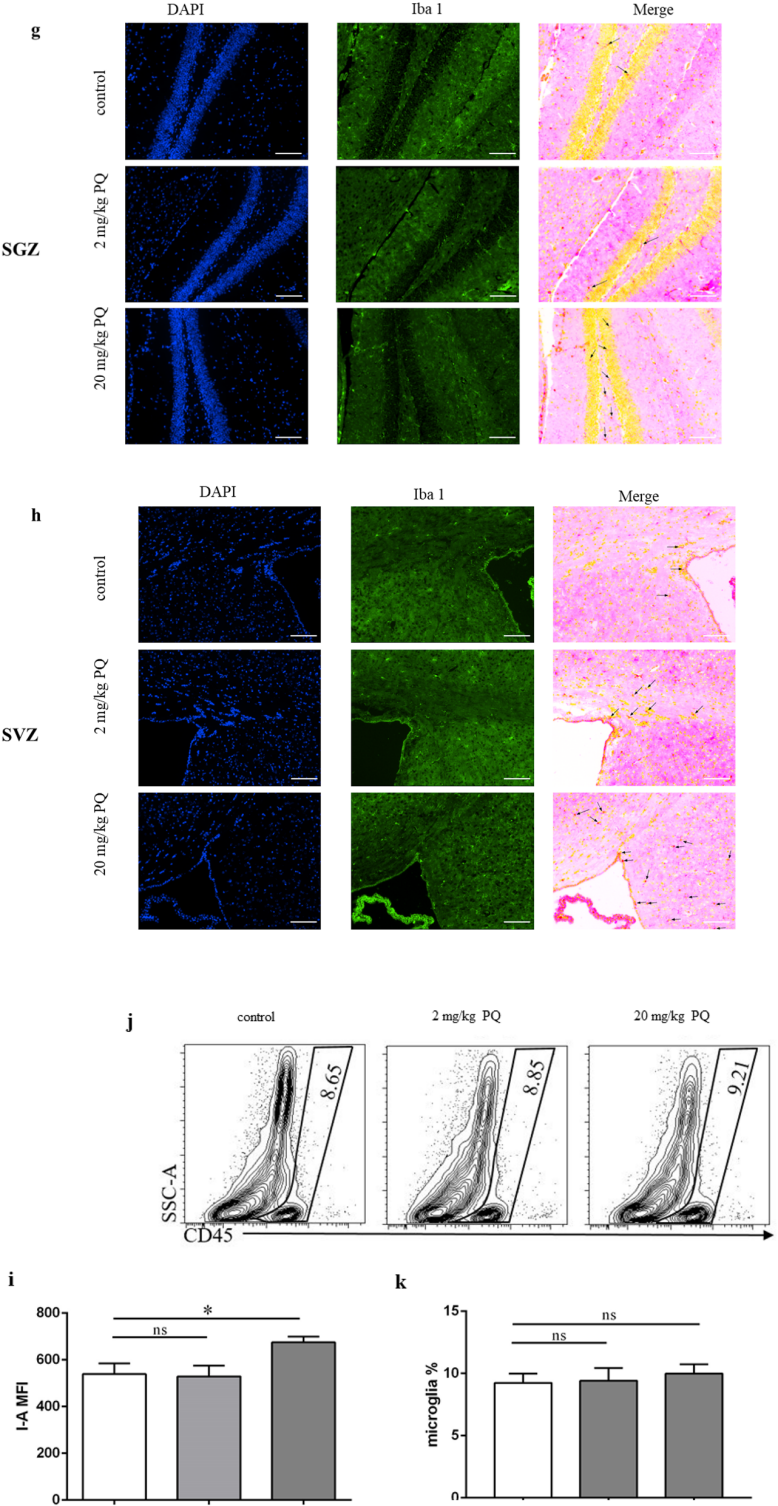
PQ caused microglia activation and induced neuroinflammation in SGZ and SVZ. The inflammatory cytokines including IL-6, IL-1β and TNF-α in SGZ and SVZ were quantified with ELISA. The expressions of CD11b and Iba1 in SGZ and SVZ were assayed by western blots. The Iba1 in SGZ and SVZ was detected by immunohistochemistry. **a**, **b** The concentrations of IL-6 (left), IL-1β (middle) and TNF-α (right) in SGZ (**a**) and SVZ (**b**). **c**, **d** The expression of the CD11b (**c**) and Iba1 (**d**) in SGZ and SVZ. The whole blots are presented in Supplementary Fig. 1. β-actin was used as internal control. **e**, **f** The quantification of CD11b (**e**) and Iba1(**f**) in SGZ. **g**, **h** Representative confocal images of Iba1 (DAPI^+^Iba1^+^) in SGZ (**g**) and SVZ (**h**); Iba1 and DAPI were in green and blue, respectively; black arrow marked Iba1^+^ cells; scale bars = 50 μm. **i**: Surface expression of I-A (MFI) on microglia. **j**, **k** Representative flow plots and quantification of microglia (CD45^+^) in the brain. Asterisk (*) indicates a significant difference (*p < 0.05, one-way ANOVA) and the n.s. means no difference as indicated.

PQ-induce neuroinflammation in mice have been widely discussed previously. Specially, we wanted to know whether the inflammation happened in SGZ and SVZ after PQ exposure in our study. Therefore, we measured the level of inflammatory cytokines, including IL-6, IL-1β and TNF-α, in both zones by ELISA. As predicted, 20 mg/kg PQ significantly increased the level of IL-6, IL-1β, and TNF-α in SGZ and SVZ (Fig. 3a, b), indicating neuroinflammation indeed happened in SVZ and SGZ where the NSC were mainly present. As microglia played a vital role in neuroinflammations diseases^28^, we next measured the activation of microglia in SGZ and SVZ by western blots, based on their surface expression of CD11b, and their cytoskeletal protein Iba1 (Fig. 3c, d). The results revealed that the expression of CD11b and Iba1 in SGZ from mice treated with 20 mg/kg PQ was significantly increased. But, limited by the biological characteristics of SVZ, the total protein levels in SVZ were too low to detect a target protein. We failed to evaluate the expression of CD11b and Iba1 in SVZ. The quantification of CD11b and Iba1 in SGZ were showed in Fig. 3e, f. To confirm the Iba1 increase, we further detected the microglia in SGZ and SVZ by immunohistochemistry staining. As expected, the Iba1 (marked by black arrow) in the mice treated with 20 mg/kg PQ had increased in both zones (Fig. 3g, h). Moreover, using flow cytometry, we quantified the microglia by CD45 antibody^29^, and evaluated the activation of microglia by detecting the expression of the Major Histocompatibility Complex class II molecules (MHC-II; I-A). As shown in Fig. 3i–k, 20 mg/kg PQ did not influence the percentage of microglia in the brain, but it resulted in the activation of microglia.

### PQ resulted in increased ER stress in microglia

**Figure 4:**
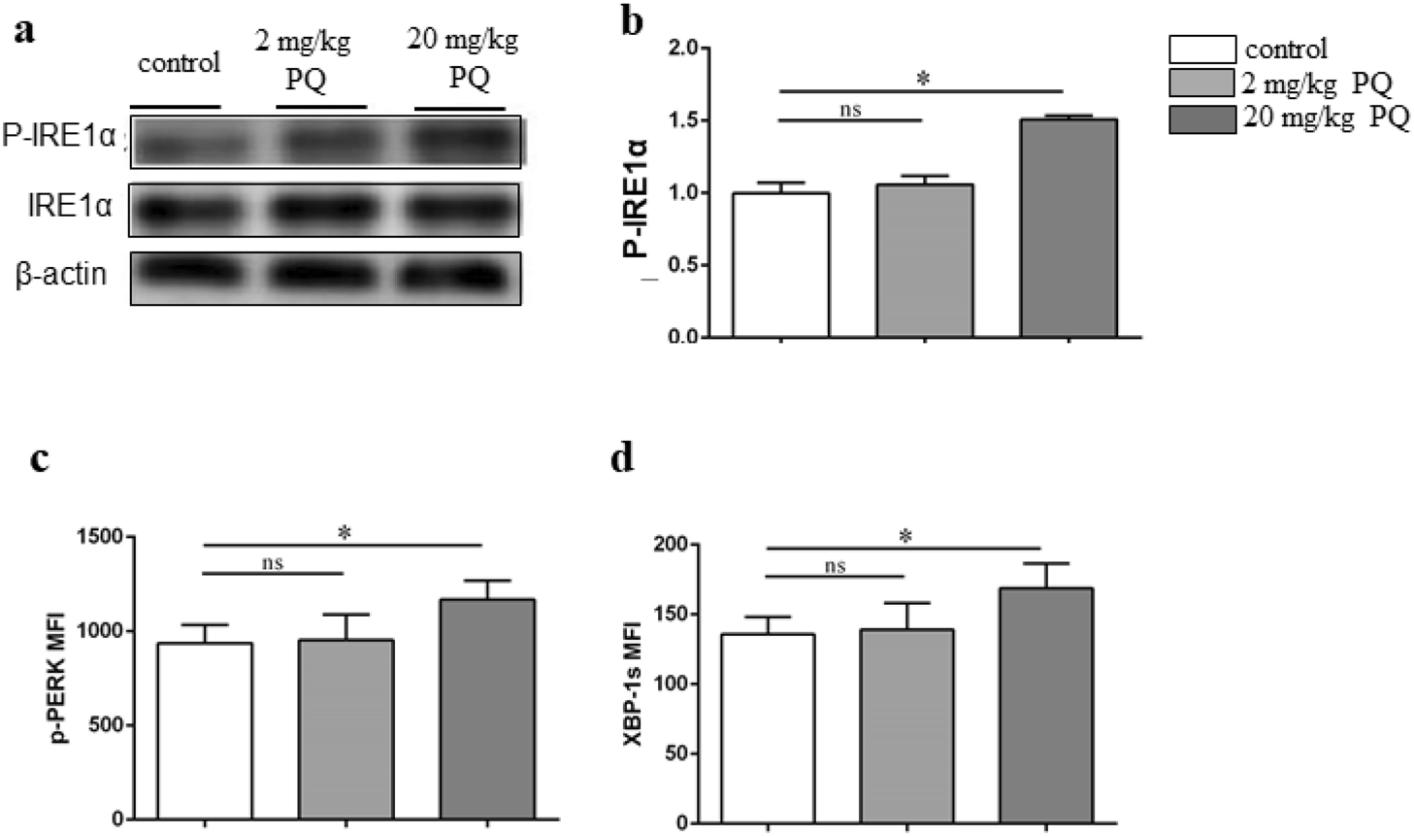
PQ resulted in increased ER stress in microglia. B6 mice were treated with PQ twice with 1wk part and thereafter the ER stress was evaluated with the western blots and flow cytometry. **a**: The expression of the p-IRE1α and total IRE1α in SGZ were assayed by western blots. The presented blots were cropped from the different membranes. Full-length blots are presented in Supplementary Fig. 2. β-actin was used as internal control. **b**: The graphs depict the p-IRE1α as showed in (**a**). **c**, **d** The expression of p-PERK (MFI) (**c**) and XBP-1 s (MFI) (**d**) in microglia (CD45^+^ cells) in SGZ upon flow cytometry analysis. Asterisk (*) indicates a significant difference (*p < 0.05, one-way ANOVA) and the n.s. means no difference as indicated. 6 mice were used for each group.

It was reported that ER stress and microglia played a vital role in inflammation-related diseases^30^. ER stress is mainly composed of three fundamental branches, including IRE1, PERK and XBP-1s. First, using western blots, we measured the activated IRE1α (p-IRE1α) in SGZ but not SVZ because of the low protein levels in SVZ. The results showed that in comparison with controls, the p-IRE1 was remarkably higher in mice treated with 20 mg/kg PQ (Fig. 4a, b). Then, using flow cytometry, we found that 20 mg/kg PQ also raised the expression of p-PERK and XBP-1 s of microglia (CD45^+^cells) in SGZ (Fig. 4c, d).

### TUDCA alleviated the neuroinflammation and NSC impairments

**Figure 5:**
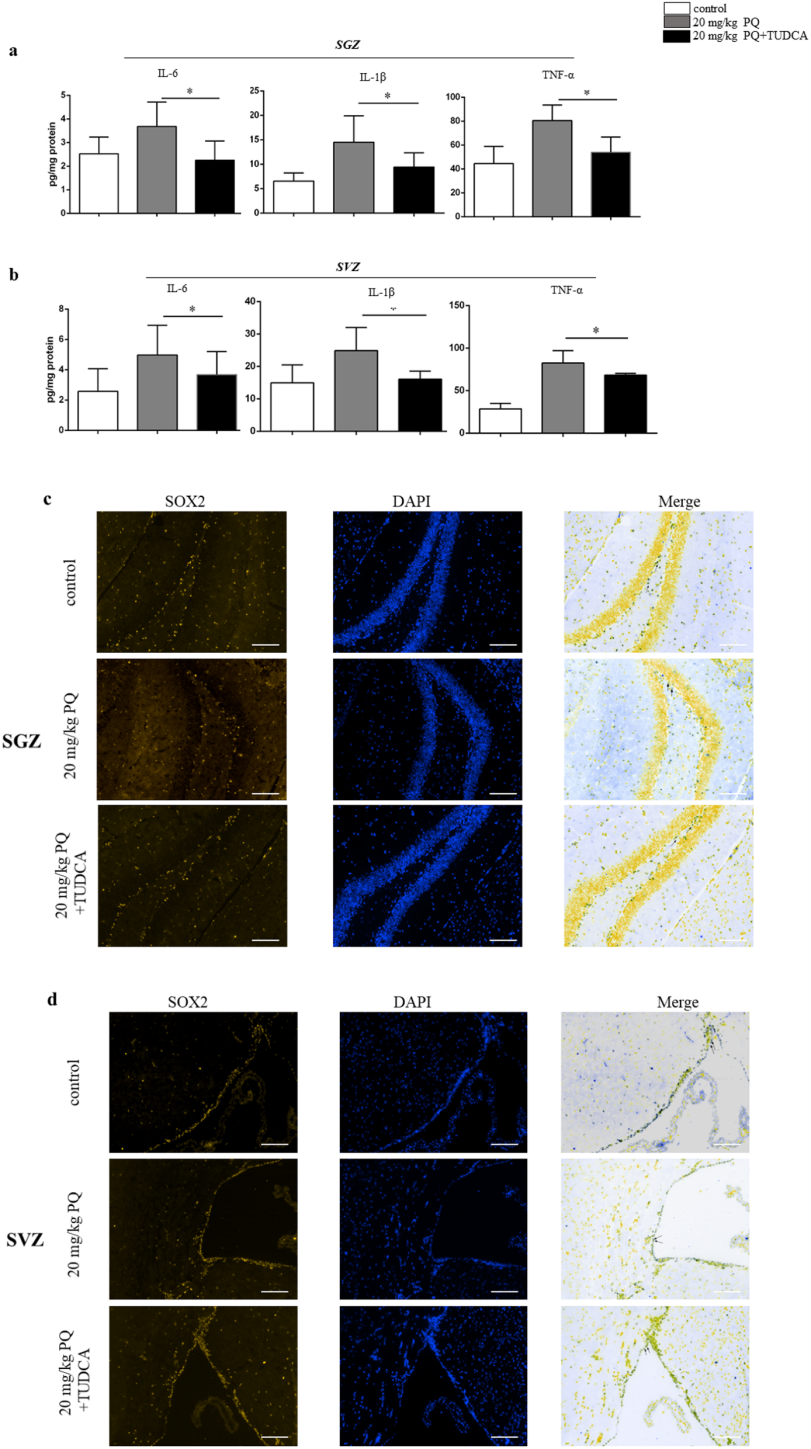

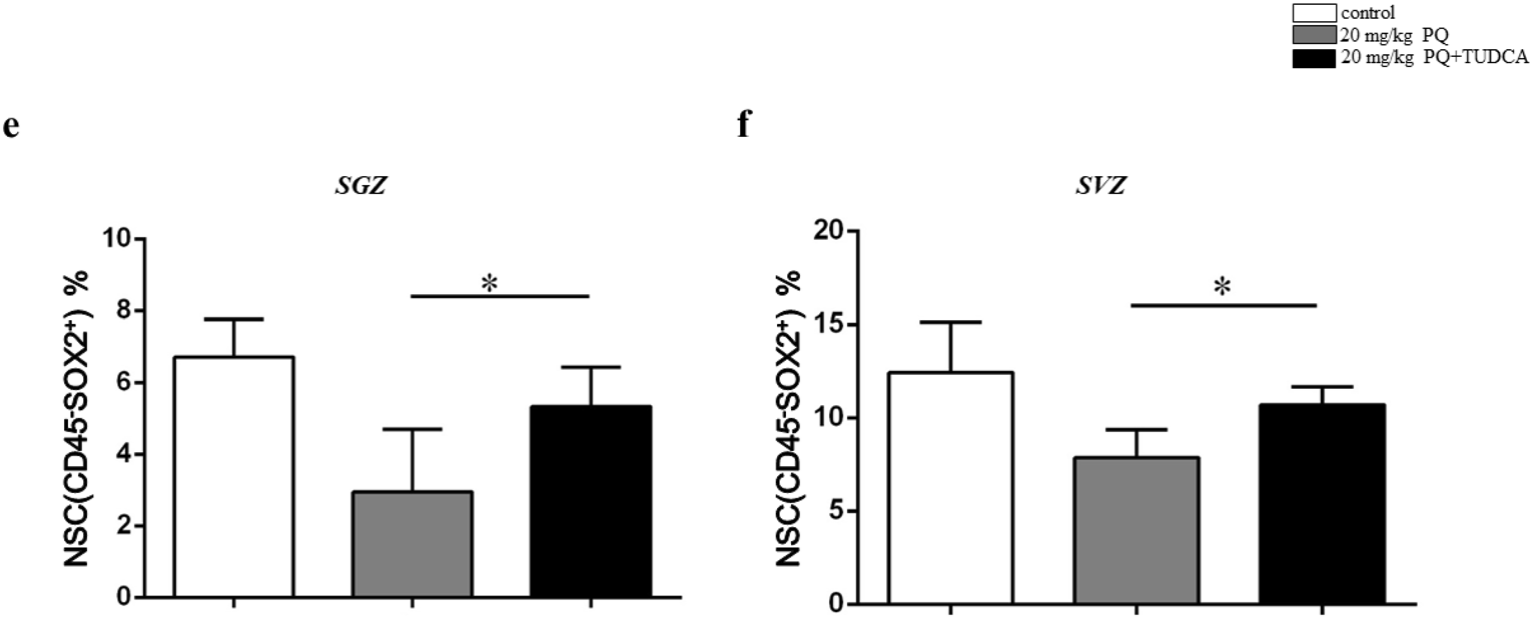
TUDCA alleviated the neuroinflammation and NSC impairments. B6 mice were treated with vehicle control, 20 mg/kg PQ or 20 mg/kg PQ + TUDCA; the inflammation cytokines were detected by ELISA; the NSCs were measured by flow cytometry and immunohistochemistry. **a**, **b** The concentrations of IL-6 (left), IL-1β (middle) and TNF-α (right) in SGZ (**a**) and SVZ (**b**). **c**, **d** Representative confocal images of NSC (DAPI^+^SOX2^+^) in SGZ (**c**) and SVZ (**d**); DAPI and SOX2 were in blue and yellow, respectively; the representative images of NSC were marked by the black arrows; all the black-green dots represent the NSC; scale bars = 50 μm. **e**, **f** The percentages of NSC (CD45^−^SOX2^+^ cells) in SGZ (**e**) and SVZ (**f**). Asterisk (*) indicates a significant difference (*p < 0.05, one-way ANOVA) and the n.s. means no difference as indicated. 6 mice were used for each group.

Inflammation could be induced by ER stress and microglia^30^. As the increased ER stress and neuroinflammation simultaneously coexisted in mice treated with 20 mg/kg PQ, we applied TUDCA to suppress the ER stress. As shown in Fig. 5a, b, the pro-inflammatory cytokines, IL-6, IL-1β and TNF-α of SGZ and SVZ in mice with 20 mg/kg PQ treatments were obviously declined after TUDCA treatments. Moreover, using histological experiments, we found that the NSC impairments were restored by TUDCA (Fig. 5c, d). Then, we further quantified the NSC by flow cytometry, and found the NSC impairments were alleviated (Fig. 5e, f).

## Discussion

Due to the severe impairment on nervous system, the PQ neurotoxicity is still a hot topic in public health and medicine. Though various in vitro studies reported that PQ elicits brain dysfunctions, such as degenerative diseases, their underlying mechanism of action, under in vivo experimental settings is not done so far^6,7^. And most of the mechanism researches were based on in vitro cell experiments. In our study, we focused on SGZ and SVZ where the NSCs persist in the adult brain, and found that 20 mg/kg PQ caused the NSC impairment. According to previous in vitro experiments, PQ could directly inhibit the viability of neural progenitor cells due to weakened proliferation or induced oxidative stress^15,31–33^. Identically, we indeed found that 20 mg/kg PQ treatment could decrease the number of NSC in SGZ and SVZ. Further analysis indicated that the NSC impairments were due to the suppressed proliferation of NSC. Although some studies suggested that PQ might increase the apoptosis of cells^4^, no increased apoptosis was observed in our study. It seems that various factors, including the dose of PQ and time of PQ exposure, may significantly influence the PQ effect^34^. The highest dose of PQ in our study was 20 mg/kg^35^, slightly higher than other studies^36^, but the mice in our study were injected with PQ only twice with 1 wk apart.

As many studies have shown that neuroinflammation could disrupt the proliferation and differentiation of NSC^20,37^, we especially focused on the inflammation that happened in SGZ and SVZ. The pro-inflammatory cytokines in SGZ and SVZ, including IL-6, IL-1β, TNF-α, were significantly increased in mice treated with 20 mg/kg PQ, indicating that the neuroinflammation indeed occurred. Previously, others and we reported that PQ-induced damages of brain function were associated with neuroinflammation, increased ROS and mitochondrial dysfunction^14,38–40^. Upon environmental stresses, neuroinflammation could be induced by microglia and/or astrocytes which were regarded as the main pro-inflammatory cytokines secreting cells in the brain^28^. As expected, we observed that microglia activation coexisted with inflammation in the brain under 20 mg/kg PQ exposure. This result was consistent with various studies, showing that PQ-induced microglia activation resulted in neuroinflammation^41–43^. The increase of ER stress induced by PQ was reported in previous researches^44–47^. In our study, we found that the PQ-increased ER stress not only occurred in the brain, but also in microglia. Interestingly, when we suppressed the ER stress using TUDCA, the neuroinflammation and NSC impairments were alleviated. But, limited by the biological characteristics of SVZ, we only detected the ER stress and microglia in SGZ, which is a deficiency in our work. Another deficiency was the selectivity and specifically of TUDCA in inhibitory on ER stress^48–51^. Collectively, those results suggested that the increased ER stress in microglia might be a possible pathway for PQ-induced neuroinflammation and NSC impairment.

## Materials and methods

### Animals

6 to 8-week (wk) old wild type (WT) C57BL/6 (B6) mice were purchased from Shanghai SLAC Laboratory Animal Co. LTD, China. Both males and females were equally used. Mice were housed in a specific pathogen free environment at Fudan University with constant temperature (23 °C) and humidity (50%) and an artificial 12-hour (hr)-light/12-hr-dark cycle. 6 mice were housed per cage with free access to food and water. The animal experiment was approved by the Ethics Committee of Animal Use at Fudan University. All methods were carried out in accordance with the guideline National Standard GB/T 35892 for Laboratory Animal Procedures of China.

### PQ treatment

PQ was purchased from Sigma, St. Louis, MO, USA. Mice were subcutaneously injected with phosphate buffered saline (PBS), 2 mg/kg PQ or 20 mg/kg PQ once a week for 2 weeks. Due to the quality of PQ in high metabolic rate, we performed preliminary experiment and found that neuroinflammation caused by PQ could persist for at least 1 wk. To further study the relationship between neuroinflammation and NSC impairments, the mice were sacrificed 1 wk after the last PQ injection.

In the ER stress function test, mice with 20 mg/kg PQ treatments were subcutaneously injected with 500 mg/kg TUDCA (APExBIO, Houston, Texas, USA) once every 3 days during PQ exposure. Then, the slices of SGZ and SVZ were prepared to measure the number of NSC.

### Flow cytometry

The SGZ and SVZ were isolated from the brain after intracardiac perfusion with 20 ml of ice-cold PBS, then digested in the trypsin and ethylenediaminetetraacetic acid (EDTA) at 37 °C for 30 min (min). The specific process of obtaining SVZ was as follows: Transect the brain about 1/2, keep the forebrain (near olfactory bulb end), and divide it into two halves along the midline. Put the lateral side of the hemi-brain down and fix it with tweezers. Use another tweezer to remove the remaining hippocampus and the inner wall of the SVZ. The outer wall of the SVZ is crescent-shaped, connected to the white matter on the upper and lower sides, with clear boundaries. The specific process of obtaining SVZ is as follows: Use forceps to separate the mouse brain, the hippocampus is located at the bottom of the cerebral cortex. Use tweezers to dislodge the cerebral cortex to expose the hippocampus. Separate hippocampal tissue from cerebral cortex and surrounding brain tissue.

Simple wash buffer (SWB), comprised by PBS and 5% fetal bovine serum (FBS) was used to stop digestion. After filtered, single cell suspensions were prepared with SWB. Antibodies (clone) and fluorochromes included V500 anti-CD45.2 (104), FITC anti-I-A (G155-178), unconjugated anti-CD16/32 (Fc block, 2.4G2) (BD Biosciences, San Jose, CA, USA); APC-Cy7 anti-CD45.2 (104), PE anti-SOX2 (14A6A34), FITC anti-Ki67 (16A8), PE anti-CD45 (30-F11), anti-Iba1(10904), anti-CD11b (66519-1-Ig), anti-Ki67 (D3B5), PE anti-XBP-1s (Q3-695) (Biolegend, San Diego, CA, USA); Alexa Fluor 488-labeled goat anti-rabbit IgG (A0423), Cyc3-labeled goat anti mouse IgG (A0521) (Beyotime, China); Alexa Fluor 488 anti-p-PERK, FITC anti-cleaved Caspase-3, FITC anti-Bcl-2 (BCL/10C4) (eBioscience, San Diego, CA, USA); and 4′, 6-diamidino-2-phenylindole (DAPI, Sigma, St. Louis, MO, USA). Cells were quantified by an automatic cell counter. Surface staining were incubated with fluorochrome-conjugated antibodies on ice in the dark for 30 min after Fc block which were performed on ice for 20 min. The intracellular and nuclear staining kits were obtained from BD Biosciences and performed following its protocol. The stained cells were analyzed with BD LSRFortessa (BD, New York, USA) and data were analyzed with FlowJo9.3.2.

### Immunohistochemistry

After fixed with 4% paraformaldehyde and dehydration with 30% sucrose solution (Sakura Finetek, Florida, USA), the brains were sliced (8 μm) and washed with PBS for 10 min, then permeabilized with 0.1 Triton X-100 (Beyotime Biotechnology, Jiangsu, China). After that, the brain slices were blocked by 5% goat serum at room temperature for 2 hr. (Santa Cruz, California, USA). The slices were incubated with SOX2 antibody at 4 °C overnight and were stained with DAPI at RT for 5 min followed by confocal analyzation. All the antibodies were purchased from Santa Cruz, California, USA. The stained slices were analyzed with confocal microscope (Nikon, Tokyo, Japan).

### Enzyme-linked immunosorbent assay (ELISA)

The cytokines IL-1β, TNF-α and IL-6 in SGZ and SVZ were detected by commercial ELISA kits, following by the manufacturer’s instructions (Biolegend, San Diego, CA, USA). The total protein concentration was quantified with BCA protein assay kit (Thermo Scientific, San Jose, CA, USA), and evaluated as pg cytokine/mg protein.

### Western blot analyses

To prepare homogenate protein, the SGZ were lysed with radio immunoprecipitation assay (RIPA) buffer containing 1% protease inhibitors (Beyotime Biotechnology, Jiangsu, China). Next, performed electrophoresis using SDS-PAGE gels and transferred the protein to polyvinylidene difluoride membranes (Millipore Corporation, Billerica MA, USA). Then, the membranes were blocked in TBST containing 5% skimmed milk powder (Sangon Biotech, Shanghai, China) at RT for 2 hr and following incubation with primary antibodies to IRE1α or phosphorylated IRE1α (p-IRE1α) at 4 °C overnight and horseradish peroxidase (HRP)-conjugated at RT for 2 hr to detect target antibody. Then ECL Western Blotting Substrate (Pierce Biotechnology, Rockford, USA) was used to visualize and the images were analyzed with ImageJ gel analysis software (NIH, USA) to quantify the protein. All the antibodies were purchased from Abcam, Cambridge, UK and β-actin were used as internal loading control.

### Statistical analyses

As no differences were observed between male and female, data were pooled and presented as mean ± standard deviation. Student’s t-tests or one-way analysis of variance (ANOVA) were performed and post-hoc LSD t-tests were used if necessary. *p* < 0.05 was considered as the level of significant difference.

## Supplementary information

Supplementary Figures.

## Data Availability

All the data in this article are available.
