# Supplementary material for: Endoplasmic reticulum stress-related neuroinflammation and neural stem cells decrease in mice exposure to paraquat
Source: Sci Rep. 2020 Oct 20;10:17757. doi: 10.1038/s41598-020-74916-x (PMC7576831; doi:10.1038/s41598-020-74916-x)

Endoplasmic reticulum stress-related neuroinflammation and neural stem cells decrease in mice exposure to paraquat

Zhengli Yang[1#], Yiming Shao[1#], Yifan Zhao[1], Qian Li[1], Rui Li[1,2], Hongxi Xiao[1], Fen Zhang [1], Yilan Zhang[3], Xiuli Chang[1], Yubin Zhang[1*] and Zhijun Zhou[1*]

[1] School of Public Health /MOE Key Laboratory of Public Health Safety/ NHC Key Lab of Health Technology Assessment, Fudan University, Shanghai 200032, China

[2]Pharmacology and Toxicology Department, Shanghai Institute for Food and Drug Control, Shanghai 201203, China

[3]Institutes of Brain Science, State Key Laboratory of Medical Neurobiology, Fudan University, Shanghai 200032, China

[#]These authors contribute equally.

***Correspondent Author**:

Yubin Zhang

School of Public Health, Fudan University

130 Dong'an Road, 8-237, Shanghai 200032, China

Phone: 86-21-54237086

Email: yz001@fudan.edu.cn

Zhijun Zhou

School of Public Health, Fudan University

130 Dong'an Road, 8-225, Shanghai 200032, China

Phone: 86-21-54237675

Email: zjzhou@fudan.edu.cn

Supplementary Figure 1

a: the representative blot for CD11b was chosen from (a)

b: the representative blot for Iba1 was chosen from (b)

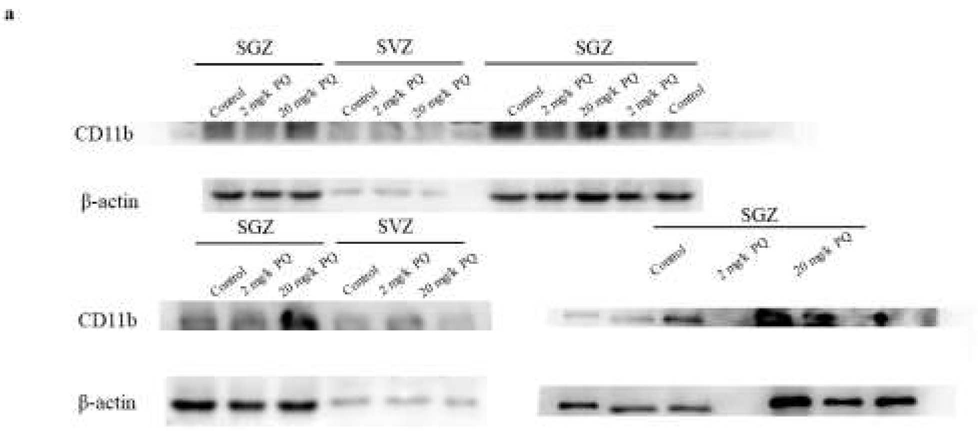

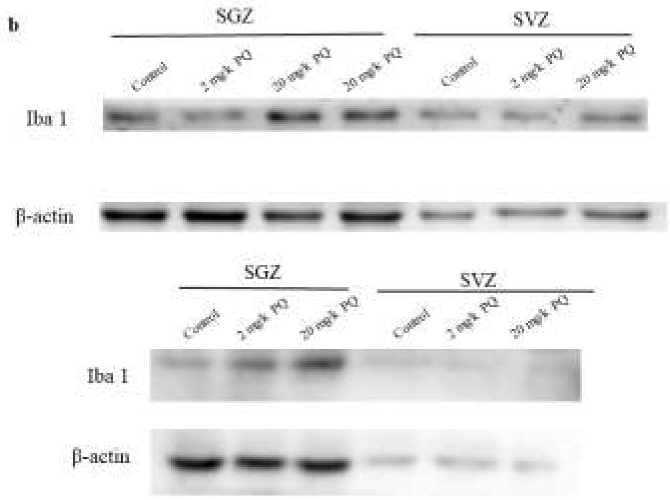

The blots presented in fig.4 were cropped from different membranes showed in Supplementary Fig.2(a-c). The representative blot was chosen in every three blots of per group.

Supplementary Figure 2

a: the full-length blot of β-actin; **b**: the full- length blot of IRE1; **c**: the full- length blot of p-IRE1.

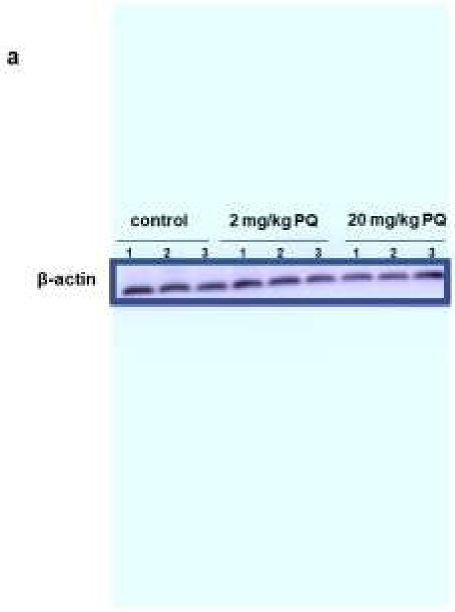

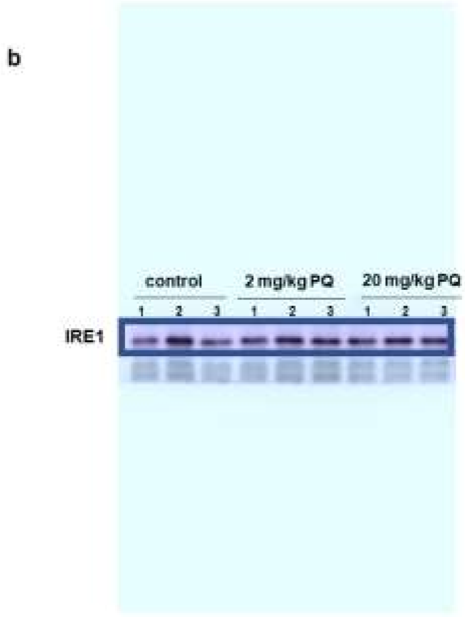

c

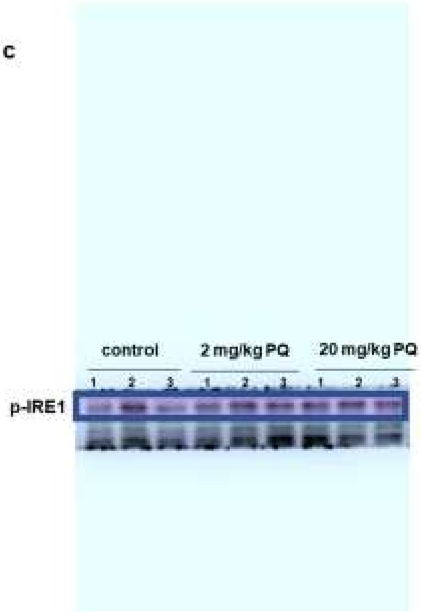

Supplement: Supplementary file 1 — Supplementary Figures. [file 41598_2020_74916_MOESM1_ESM.pdf]
